# Prediction of protein secondary structures with a novel kernel density estimation based classifier

**DOI:** 10.1186/1756-0500-1-51

**Published:** 2008-07-23

**Authors:** Darby Tien-Hao Chang, Yu-Yen Ou, Hao-Geng Hung, Meng-Han Yang, Chien-Yu Chen, Yen-Jen Oyang

**Affiliations:** 1 Department of Electrical Engineering, National Cheng Kung University, Tainan, 70101, Taiwan, ROC; 2 Graduate School of Biotechnology and Bioinformatics, Yuan Ze University, Chung-Li, 320, Taiwan, ROC; 3 Department of Computer Science and Engineering, Yuan Ze University, Chung-Li, 320, Taiwan, ROC; 4 Department of Computer Science and Information Engineering, National Taiwan University, Taipei, 106, Taiwan, ROC; 5 Department of Bio-Industrial Mechatronics Engineering, National Taiwan University, Taipei, 106, Taiwan, ROC; 6 Graduate Institute of Biomedical Electronics and Bioinformatics, National Taiwan University, Taipei, 106, Taiwan, ROC; 7 Graduate Institute of Networking and Multimedia, National Taiwan University, Taipei, 106, Taiwan, ROC; 8 Center for Systems Biology and Bioinformatics, National Taiwan University, Taipei, 106, Taiwan, ROC

## Abstract

**Background:**

Though prediction of protein secondary structures has been an active research issue in bioinformatics for quite a few years and many approaches have been proposed, a new challenge emerges as the sizes of contemporary protein structure databases continue to grow rapidly. The new challenge concerns how we can effectively exploit all the information implicitly deposited in the protein structure databases and deliver ever-improving prediction accuracy as the databases expand rapidly.

**Findings:**

The new challenge is addressed in this article by proposing a predictor designed with a novel kernel density estimation algorithm. One main distinctive feature of the kernel density estimation based approach is that the average execution time taken by the training process is in the order of *O*(*n*log*n*), where *n *is the number of instances in the training dataset. In the experiments reported in this article, the proposed predictor delivered an average Q_3 _(three-state prediction accuracy) score of 80.3% and an average SOV (segment overlap) score of 76.9% for a set of 27 benchmark protein chains extracted from the EVA server that are longer than 100 residues.

**Conclusion:**

The experimental results reported in this article reveal that we can continue to achieve higher prediction accuracy of protein secondary structures by effectively exploiting the structural information deposited in fast-growing protein structure databases. In this respect, the kernel density estimation based approach enjoys a distinctive advantage with its low time complexity for carrying out the training process.

## Findings

### Motivation

In structural biology, protein secondary structures act as the building blocks for the protein tertiary structures [1,2]. Therefore, analysis of protein secondary structures is an essential intermediate step toward obtaining a comprehensive picture of the tertiary structure of a protein. In this respect, one of the main challenges is how to accurately identify the polypeptide segments that could fold to form a secondary structure. This problem is normally referred to as prediction of protein secondary structures [1,3].

Though prediction of protein secondary structures has been an active issue in bioinformatics research for quite a few years and many approaches have been proposed [1,4-10], a new challenge emerges as the sizes of contemporary protein structure databases continue to grow rapidly. The new challenge, which has been addressed in several recently completed studies [9-11], is concerned with how we can effectively exploit the information implicitly deposited in the ever-growing protein structure databases and deliver ever-improving prediction accuracy. In this respect, this article proposes the Prote2S predictor designed with a novel kernel density estimation algorithm [12], which features an average time complexity of *O*(*n*log*n*) for carrying out the training process, where *n *is the number of instances in the training dataset.

### Experimental results

This section reports the experiments conducted to investigate how Prote2S performs in comparison with the other existing predictors of protein secondary structures. The design of Prote2S is based on the relaxed variable kernel density estimator (RVKDE) that we have recently proposed [12]. In the next section, we will discuss how the RVKDE has been incorporated in the design of Prote2S and the related issues.

For Prote2S, the training dataset was derived from the PDB version available at the end of May, 2007. In order to guarantee that no protein chains used to generate the training dataset is homologous to the benchmark protein chains on the EVA server [13], from which the testing dataset was extracted, BLAST [14] was invoked and the criterion of homology was set to sequence identity higher than 25%. Then, the CD-HIT clustering algorithm [15] with the similarity threshold set to 0.4 was invoked to remove redundant protein chains in the PDB. After these two processes, a total of 8006 protein chains remained. To generate the training dataset, we followed the approach employed in [6]. Accordingly, one training instance was created for each residue in the 8006 protein chains by associating the residue with the position specific scoring matrix (PSSM) computed by the PSI-BLAST software package [14] with window size set to 15. As a result, a total of 1,801,039 training instances were generated and each was labeled by DSSP [16] as one of the three types of secondary structure elements: alpha-helix, beta-strand, or coil.

The testing dataset used in the following experiments was derived from the 106 benchmark protein chains released on the EVA server between September 7, 2004 and March 1, 2006. We extracted only those 89 protein chains of which the prediction results made by all the 5 predictors involved in the comparison are available on the EVA server. The testing dataset then comprises 27 long protein chains, each of which contains more than 100 residues, and 62 short protein chains.

In addition to the training and testing datasets, we generated a validation dataset for tuning the parameters in Prote2S. How the validation dataset was generated and how the validation process was carried out will be elaborated in the next section.

Table 1, 2, 3 show how Prote2S performs with the testing dataset in comparison with the other predictors whose results are available on the EVA server. In Tables 1 and 2, we report the accuracies deliver by alternative predictors with protein chains longer than 100 residues and with those shorter than 100 residues, respectively. One interesting observation is that most predictors delivered higher prediction accuracy with the long protein chains than with the short ones. Furthermore, Prote2S delivered the highest prediction accuracy with the long protein chains in comparison with the other predictors. If we use the rule-of-thumb proposed in [11], then the Q_3 _score delivered by Prote2S with long protein chains is significantly higher than those delivered by the other predictors. On the other hand, though Prote2S still leads in terms of the SOV score with long protein chains, the difference is not significant.

**Table 1:** Prediction accuracies delivered by alternative predictors with the 27 protein chains longer than 100 residues extracted from the EVA server.

	Q_3_	Q_3_H_O	Q_3_H_P	Q_3_E_O	Q_3_E_P	Q_3_C_O	Q_3_C_P	SOV	SOVH	SOVE	SOVC
Prote2S	80.3%	76.4%	78.3%	60.5%	75.8%	84.1%	76.3%	76.9%	77.7%	64.9%	75.2%
Errsig	2.0%	3.8%	3.4%	9.3%	7.8%	2.0%	2.4%	2.2%	3.2%	9.4%	2.4%
PSIPRED	78.2%	78.0%	76.4%	60.6%	67.3%	77.0%	75.3%	75.0%	76.2%	62.7%	72.0%
Errsig	1.2%	4.1%	3.8%	9.0%	9.4%	1.8%	1.9%	1.4%	3.7%	9.0%	1.8%
PROFsec	77.9%	71.6%	81.6%	61.0%	63.4%	80.2%	72.7%	76.1%	75.4%	64.1%	73.0%
Errsig	1.2%	3.7%	3.8%	9.2%	9.2%	2.0%	1.6%	1.4%	3.8%	9.2%	1.9%
PHDpsi	75.2%	76.4%	77.3%	55.5%	61.9%	74.1%	72.5%	72.5%	75.6%	56.3%	70.1%
Errsig	1.3%	3.5%	3.7%	8.8%	9.3%	2.6%	2.1%	1.7%	3.4%	8.9%	2.4%
SABLE2	77.0%	74.0%	79.3%	55.2%	75.0%	80.2%	71.4%	72.6%	74.5%	59.9%	70.1%
Errsig	1.3%	3.5%	3.1%	8.9%	4.8%	2.4%	1.7%	2.0%	3.1%	9.1%	2.6%
PROF_king	70.7%	56.6%	72.7%	55.8%	57.8%	77.6%	67.1%	67.5%	60.9%	58.6%	68.2%
Errsig	1.5%	4.6%	7.8%	9.1%	7.2%	1.8%	2.1%	1.6%	4.6%	9.1%	2.2%

Errsig is the significant difference margin for each score and is defined as the standard deviation over the square root of the number of proteins. Q_3_H/E/C and SOVH/E/C values are the specific Q_3 _and SOV scores of the predicted helix, strand and coil regions, respectively. Q_3_H_O (Q_3_E_O and Q_3_C_O, respectively) represents correctly predicted helix (strand and coil, respectively) residues (percentage of helix observed), and Q_3_H_P (Q _3_E_P and Q_3_C_P, respectively) represents correctly predicted helix (strand and coil, respectively) residues (percentage of helix predicted).

**Table 2:** Prediction accuracies delivered by alternative predictors with the 62 protein chains shorter than 100 residues extracted from the EVA server.

	Q_3_	Q_3_H_O	Q_3_H_P	Q_3_E_O	Q_3_E_P	Q_3_C_O	Q_3_C_P	SOV	SOVH	SOVE	SOVC
Prote2S	75.1%	73.1%	79.4%	69.7%	73.7%	85.3%	70.6%	69.4%	74.7%	71.8%	72.4%
Errsig	1.5%	3.5%	3.6%	4.4%	4.7%	1.6%	2.2%	2.5%	3.5%	4.3%	2.1%
PSIPRED	77.0%	78.4%	80.3%	69.8%	76.9%	77.5%	77.7%	73.2%	75.4%	72.1%	72.6%
Errsig	1.6%	3.9%	3.2%	4.3%	3.9%	1.8%	2.0%	2.2%	3.9%	4.3%	2.2%
PROFsec	76.4%	78.0%	82.4%	75.8%	69.7%	79.6%	74.0%	72.9%	79.7%	77.7%	71.0%
Errsig	1.5%	3.1%	3.2%	3.5%	4.4%	1.6%	1.9%	2.2%	3.1%	3.5%	2.3%
PHDpsi	75.6%	82.7%	76.1%	70.4%	67.5%	75.4%	77.2%	70.2%	79.4%	72.0%	69.1%
Errsig	1.7%	3.1%	3.6%	4.1%	4.7%	1.9%	1.9%	2.4%	3.3%	4.1%	2.5%
SABLE2	76.3%	76.1%	76.4%	71.3%	61.2%	80.7%	74.8%	71.5%	77.1%	72.1%	71.0%
Errsig	1.6%	3.6%	4.0%	4.1%	5.0%	1.4%	2.0%	2.3%	3.7%	4.2%	2.2%
PROF_king	72.5%	67.4%	83.5%	72.6%	66.6%	79.9%	70.1%	65.8%	67.2%	72.8%	68.5%
Errsig	1.7%	4.1%	3.3%	4.2%	4.7%	1.6%	2.3%	2.5%	4.2%	4.4%	2.4%

**Table 3:** Prediction accuracies delivered by alternative predictors with the 89 benchmark protein chains extracted from the EVA server.

	Q_3_	Q_3_H_O	Q_3_H_P	Q_3_E_O	Q_3_E_P	Q_3_C_O	Q_3_C_P	SOV	SOVH	SOVE	SOVC
Prote2S	76.7%	74.1%	79.1%	71.4%	76.6%	84.9%	72.3%	71.7%	75.6%	74.2%	73.3%
Errsig	1.3%	2.7%	2.7%	3.2%	3.5%	1.3%	1.7%	1.9%	2.6%	3.2%	1.6%
PSIPRED	77.4%	78.3%	79.1%	71.5%	78.5%	77.3%	77.0%	73.7%	75.7%	73.8%	72.4%
Errsig	1.2%	3.0%	2.5%	3.1%	2.9%	1.4%	1.5%	1.6%	2.9%	3.1%	1.6%
PROFsec	76.9%	76.0%	82.1%	75.8%	72.3%	79.7%	73.6%	73.9%	78.4%	78.0%	71.6%
Errsig	1.1%	2.5%	2.5%	2.6%	3.2%	1.3%	1.4%	1.6%	2.5%	2.6%	1.7%
PHDpsi	75.5%	80.8%	76.5%	70.4%	70.3%	75.0%	75.8%	70.9%	78.2%	71.7%	69.4%
Errsig	1.3%	2.4%	2.7%	3.0%	3.4%	1.5%	1.5%	1.7%	2.5%	3.0%	1.9%
SABLE2	76.5%	75.5%	77.3%	70.9%	65.4%	80.6%	73.7%	71.8%	76.3%	72.9%	70.7%
Errsig	1.2%	2.7%	2.9%	3.0%	3.8%	1.2%	1.5%	1.7%	2.7%	3.1%	1.7%
PROF_king	72.0%	64.1%	82.5%	72.0%	66.2%	79.2%	69.1%	66.3%	65.3%	73.0%	68.4%
Errsig	1.2%	3.2%	2.6%	3.1%	3.5%	1.2%	1.7%	1.8%	3.3%	3.2%	1.8%

Though the prediction accuracy delivered by Prote2S with long protein chains is superior, Prote2S did not perform as well with short protein chains. In fact, the prediction accuracy delivered by Prote2S with short protein chains is inferior to most predictors listed in Table 2. Accordingly, we can conclude that alternative machine learning algorithms offer different advantages and suffer some limitations. Therefore, it may be desirable to design a hybrid predictor that exploits the respective advantages of alternative predictors. For example, we may implement a hybrid predictor that invokes Prote2S when dealing with a long protein chain and invokes PSIPRED otherwise.

As mentioned earlier, one of the major distinctive feature of the RVKDE-based predictor is that the average time taken to construct a predictor is in the order of *O*(*n*log*n*), where *n *is the number of training instances. Therefore, it is conceivable that Prote2S can effectively cope with the high growth rate of the PDB and deliver ever-increasing prediction accuracy. In this respect, the experiment reported in Table 4 has been conducted to evaluate the related effects. In this experiment, we provided Prote2S and the LIBSVM package [17] with randomly generated subsets of the training dataset and testing was conducted with the 27 long protein chains in the testing dataset. The Gaussian kernel was adopted in LIBSVM and the two related parameters were set as C = 2 and *γ *= 0.01 based on the model selection process employed in [18]. The execution times shown in Table 4 were measured on a workstation equipped with an Intel Xeon 3.2GHz CPU and 8-GByte memory and do not include the time taken to carry out model selection or cross validation.

**Table 4:** Size of the training dataset vs. execution times taken by the Prote2S and the SVM during the training process.

	Prote2S			SVM		
		
Number of protein chains used to generate the training dataset	Training time (in seconds)	Q_3_	SOV	Training time (in seconds)	Q_3_	SOV
50	29.6	64.0%	52.9%	138.08	71.3%	64.3%
100	91.7	69.0%	64.1%	527.02	74.0%	68.3%
250	486.4	71.4%	67.2%	5105.63	75.5%	71.0%
500	1377.4	71.9%	67.9%	21040.0	76.8%	72.3%
1000	3887.8	73.9%	71.1%	78795.25	77.4%	73.3%

The first observation about the experimental results presented in Table 4 is that the training time with the LIBSVM increases approximately in the order of *O*(*n*^2^). On the other hand, the training time with the Prote2S increases approximately in the order of *O*(*n*log*n*). Accordingly, it is conceivable that simply employing the SVM might be impractical for some bioinformatics applications, in which the database involved is already large and still growing fast. Another observation with Table 4 is that LIBSVM generally delivered higher prediction accuracy than Prote2S but the difference diminishes as the size of the training dataset increases. This observation is consistent with that reported by the research team led by D.T. Jones [6]. According to their study, the SVM can deliver higher prediction accuracy than a neural network when the training dataset is small and the difference diminishes as the size of the training dataset increases.

Our proposition concerning the inferior accuracies delivered by Prote2S in Table 4 is that it results from the asymptotic approach employed to establish the mathematical foundation of kernel density estimation [12,19]. Since the asymptotic approach assumes that the number of training instances approaches infinity, under circumstances in which the size of the training dataset is not sufficiently large, the mathematical model of a kernel density estimator may become inaccurate and the kernel density estimation based predictor may deliver inferior accuracy. Nevertheless, as the size of the training dataset increases, this effect should diminish.

Another aspect of the execution time with a predictor is the time taken to make a prediction. In this respect, it has been shown in our recent article that the average time taken by the RVKDE-based predictor to make predictions with *n' *incoming objects is in the order of *O*(*n' *log *n*) [12]. Table 5 shows how the execution times taken by Prote2S and LIBSVM to make predictions increase with the size of the training dataset. The results show that the execution time taken by Prote2S increases slower than that taken by the SVM, which grows approximately in the same order as the size of the training dataset. In this experiment, we provided Prote2S and the LIBSVM package [17] with randomly generated subsets of the training dataset and testing was conducted with the 27 long protein chains in the testing dataset.

**Table 5:** Size of the training dataset vs. execution times taken by Prote2S and the SVM for making predictions.

	Prote2S	SVM
		
Number of protein chains used to generate the training dataset	Testing time (in seconds)	Testing time (in seconds)
50	54.5	146.7
100	87.6	301.0
250	153.3	758.5
500	220.7	990.7
1000	333.2	2532.8

### The RVKDE based predictor

As mentioned above, the design of Prote2S is based on a novel kernel density estimation algorithm. The mathematical fundamentals of the so-called RVKDE can be found in our recent publication [12]. A kernel density estimator is in fact an approximate probability density function. Let {***s*_1_**, ***s*_2_**..., ***s*_*n*_**} be a set of sampling instances randomly and independently taken from the distribution governed by *f*_*X *_in the *m*-dimensional vector space. Then, with the RVKDE algorithm, the value of *f*_*X *_at point ***v ***is estimated as follows:

$$\hat{f}(v)=\frac{1}{\left|n\right|}\sum_{s_{i}}{\left(\frac{1}{\sqrt{2\pi }\cdot \sigma_{i}}\right)}^{m}\exp\left(-\frac{||v-s_{i}|{|}^{2}}{2\sigma_{i}^{2}}\right),\text{ where}$$

1) $\sigma_{i}=\beta \frac{R(s_{i})\sqrt{\pi }}{\sqrt{\left(k+1\right)\Gamma \left(\frac{m}{2}+1\right)m}}$;

2) *R*(***s*_*i*_**) is the maximum distance between ***s*_*i *_**and its *k *nearest training instances;

3) Γ (·) is the Gamma function [20];

4) *β *and *k *are parameters to be set either through cross validation or by the user.

For prediction of protein secondary structures, one kernel density estimator is created to approximate the distribution of each class of training instances. As mentioned earlier, in our experiment, each residue is associated with a PSSM computed with the PSI-BLAST software package, and is labeled as one of the three types of secondary structure elements: alpha-helix, beta-strand, or coil, as determined by DSSP. Then, a query instance located at *v *is predicted to belong to the class that gives the maximum value with the likelihood function defined as follows:

$$L_{j}(v)=\frac{\left|S_{j}|\cdot {\hat{f}}_{j}(v\right)}{\sum_{h}\left|S_{h}|\cdot {\hat{f}}_{h}(v\right)},$$

where |*S*_*j*_| is the number of class-*j *training instances, and ${\hat{f}}_{j}(v)$ is the kernel density estimator corresponding to class-*j *training instances. In our current implementation, in order to improve the efficiency of the predictor, we include only a limited number, denoted by *k'*, of the nearest class-*j *training instances of ***v ***while computing ${\hat{f}}_{j}(v)$.

With the predictions made by the RVKDE based algorithm for the query protein chain, Prote2S carries out a smoothing process as the last step before outputting the results. The smoothing process includes two phases. In the first phase, each single-residue segment of secondary structures with its two neighboring residues belonging to the same secondary structure is examined to determine whether switching the prediction of the single-residue segment to the same secondary structure as its neighbors can form a new segment containing 4 or more residues. If yes, then the switching is carried out. Otherwise, nothing will happen. In the second phase, all the remaining single-residue segments of secondary structures except those predicted to be a coil are located and the prediction of each segment is switched to the secondary structure of its longer neighboring segment.

### Parameter tuning

In the experiments reported in this article, the 4 parameters in the RVKDE algorithm were set as *m *= 1, *β *= 3, *k *= 38, and *k' *= 60, through a validation process. The validation dataset was derived from the 1903 protein chains deposited into the PDB between June 1 and August 31 in 2007. In order to remove redundancy, BLAST was invoked to guarantee that the BLAST-computed e-value similarity score between any two protein chains in the validation dataset is larger than 0.1. Furthermore, we removed those protein chains that are homologous to one or more of the protein chains used to generate the training dataset with a BLAST-computed sequence identity larger than 25%. As a result, a total of 302 protein chains remained. Among these 302 protein chains, we then included those 45 chains that are longer than 100 residues to generate the validation dataset.

## Competing interests

The authors declare that they have no competing interests.

## Authors' contributions

YJO proposed the RVKDE algorithm and conceived the study. DTHC and YYO implemented the Prote2S predictor. HGH, MHY, and CYC designed the experiments reported in this article. All authors read and approved the final manuscript.
